# Auditory cueing modulates motor performance and brain activity in early Parkinson’s disease—a kinematic and fMRI study

**DOI:** 10.1038/s41531-026-01438-0

**Published:** 2026-06-19

**Authors:** Andrea Gardoni, Elisabetta Sarasso, Marco Forghieri, Andrea Grassi, Roberta Balestrino, Silvia Basaia, Chiara Tripodi, Elisa Canu, Maria Antonietta Volontè, Davide Corbetta, Massimo Filippi, Federica Agosta

**Affiliations:** 1 Neurotech Hub, Vita-Salute San Raffaele University, Milan, Italy; 2 Neuroimaging Research Unit, Division of Neuroscience, IRCCS San Raffaele Scientific Institute, Milan, Italy; 3 Department of Neuroscience, Rehabilitation, Ophthalmology, Genetics and Maternal Child Health, University of Genoa, Genoa, Italy; 4 Neurology Unit, IRCCS San Raffaele Scientific Institute, Milan, Italy; 5 Neurorehabilitation Unit, IRCCS San Raffaele Scientific Institute, Milan, Italy; 6 Department of Rehabilitation and Functional Recovery, IRCCS San Raffaele Scientific Institute, Milan, Italy; 7 Neurophysiology Service, IRCCS San Raffaele Scientific Institute, Milan, Italy

**Keywords:** Neurology, Neuroscience

## Abstract

Cues are specific references that support motor performance by improving speed, timing, and amplitude while reducing variability. This study aimed at investigating the effects of external auditory cueing on bradykinesia-related kinematic deficits and their neural correlates during a hand-tapping task in people with Parkinson’s disease (pwPD) and healthy controls (HC). Twenty-six pwPD and 20 HC performed a right hand-tapping fMRI task either in a self-paced condition (NOCue) or following an auditory cue (Cue). Hand-tapping kinematics were objectively quantified using an optical-fiber glove. PwPD showed reduced hand-tapping amplitude and a greater sequence effect on amplitude than HC during NOCue condition. Cueing attenuated the sequence effect on amplitude in pwPD. During the NOCue condition, pwPD showed reduced activity of pallidum/thalamus relative to HC. No group differences emerged during Cue condition. During Cue relative to NOCue condition, pwPD showed higher activity of pallidum, parietal, temporal, sensorimotor, insular, and cerebellar regions, alongside reduced activation in of paracentral gyrus and cerebellum. Higher activity of pallidum correlated with better motor performance and higher positive effects of cueing. Our results demonstrated that auditory cueing reduces bradykinesia in pwPD possibly by facilitating basal ganglia activity and reducing the need of movement programming.

## Introduction

Bradykinesia has been recently redefined to include a broader complex of motor alterations rather than the classical progressive reduction in amplitude and/or speed with the repetition of movement^1^. The “bradykinesia complex” encompasses bradykinesia, hypokinesia characterized by reduced movement amplitude, akinesia, oligokinesia defined as a reduction or absence of spontaneous or automatic movements, as well as sequence effect and hesitations or halts. This constellation of motor features reflects a network-level dysfunction involving impaired motor scaling, altered sequence generation, defective sensorimotor integration, and reduced ability to maintain movement rhythm over time^1–3^. Bradykinesia is the cardinal motor manifestation in people with Parkinson’s disease (pwPD) and a major contributor to disability in daily activities^4,5^. There is a great interest in understanding the neural correlates of this phenomenon, and in providing valid measurement tools and rehabilitation strategies^6–9^. Among rehabilitation strategies for pwPD, external cueing is widely employed. Cues are described as specific reference points aimed at supporting and enhancing motor performance^10^. They are classified into three primary modalities: visual cues, auditory cues, and somatosensory cues. The primary neurophysiological explanation for the effectiveness of external cueing is that cues promote a shift from impaired neural pathways to intact ones, effectively redirecting behavior from habitual patterns to more goal-directed actions^11^. Rhythmic auditory cues involve the connections between the auditory and motor systems by using metronomes or familiar music to deliver the external cues. The optimal frequency for spontaneous human movement typically falls within cycles of 500–600 ms, consistent with the 2-Hz human resonance theory, a phenomenon observed across behaviors such as walking and clapping and reflected in the preferred tempo of most music. This strategy has been shown to improve movement speed, timing, and amplitude, while also reducing variability in gait, as well as in upper limbs and finger movements^12^.

Functional magnetic resonance imaging (fMRI) has provided valuable insight into the altered motor circuitry in pwPD. Across investigations, differences converged in the posterior putamen, precentral gyrus, and cerebellum^13^. Studies have used a variety of tasks to assess brain activity related to movement and to the effects of cueing^14–16^. When focusing on hand movement, studies have documented aberrant activation patterns and altered connectivity in key regions for temporal processing such as supplementary motor area (SMA), basal ganglia, cerebellum, and right inferior frontal and parietal cortices in pwPD compared to healthy controls during cued tasks^17^. However, brain activity differences can reflect either true differences in neural control or simply differences in executed kinematics (e.g., slower speed, smaller amplitude, or variable movement)^9,18–20^. Simultaneous acquisition of high-resolution kinematics and fMRI addresses this matter but remains rare, limiting the interpretation of the results. Magnetic resonance-compatible devices, such as optical fiber data gloves, allow direct recording of amplitude, timing, and progressive amplitude/speed decrement during in-scanner tasks. Using this methodology, we recently compared pwPD and matched healthy controls performing hand tapping movements during fMRI^9^. PwPD showed reduced amplitude, greater sequence effect, and concurrent reductions in activation of SMA, frontoparietal cortex, thalamus/pallidum, and cerebellar motor areas^9^.

Nonetheless, most published works still treats kinematics and imaging separately or uses only gross behavioral measures, leaving a gap in our mechanistic understanding of how cueing alters both movement execution and its neural substrates^21^.

The present study addressed these gaps using an optic fiber glove to extract objective kinematic parameters during a hand tapping fMRI task to compare internally generated (self-paced) and externally cued movement in pwPD and matched healthy controls. The study aimed at determining the effects of auditory cues on motor performance and on underlying neural activity.

## Results

### Participants

Twenty-six pwPD and 20 age- and sex-matched healthy subjects were included. The two groups were similar for sociodemographic variables, except for education that was higher in healthy controls (Table 1). PwPD were in the early phase of the disease, with a mean disease duration of 3.62 ± 2.75 years and a mean MDS-UPDRS-III of 32.19 ± 10.22 during OFF medication state and of 25.46 ± 9.48 during ON state (Table 1). Relative to healthy controls, pwPD showed reduced manual dexterity (PPT) and reported a worse manual ability in daily activities (MAM-36).

**Table 1:** Sociodemographic and clinical characteristics of people with Parkinson’s disease and healthy controls

	pwPD (*N* = 26)	HC (*N* = 20)	p pwPD vs HC
**Age (years)**	66.29 ± 7.57 (52.39; 80.27)	63.63 ± 5.60 (53.71; 74.06)	0.19
**Sex (M/F)**	18/8	14/6	1.00
**Education (years)**	11.65 ± 3.70 (6; 21)	15.10 ± 2.32 (13; 18)	**<0.001**
**Disease duration (years)**	3.62 ± 2.75 (0.5; 12)	–	–
**LEDD (mg)**	421.96 ± 276.88 (50; 1090)	–	–
**UPDRS-II**	9.24 ± 5.70 (2; 23)	–	–
**UPDRS-III on**	25.46 ± 9.48 (8; 43)	.–	–
**UPDRS-III off**	32.19 ± 10.22 (15; 62)	–	–
**H&Y on (1/1.5/2/2.5)**	1/1/23/1	–	–
**H&Y off (1/2/3)**	1/24/1	–	–
**MAM-36**	93.05 ± 6.38 (77.27; 100.00)	100 ± 0.00 (100; 100)	**<0.001**
**PPT right**	12.08 ± 1.87 (7.00; 17.00)	14.58 ± 1.56 (11.67; 17.30)	**<0.001**
**PPT left**	11.59 ± 1.85 (7.33; 16.00)	13.87 ± 1.88 (11.00; 16.67)	**0.01**
**PPT bilateral**	8.89 ± 1.64 (4.67; 13.67)	11.40 ± 1.37 (9.33; 13.33)	**<0.001**
**PDQ-39**	12.72 ± 9.21 (2.62; 40.99)	–	–
**MMSE**	28.69 ± 1.67 (24; 30)	29.20 ± 0.83 (28; 30)	0.29

Values are mean ± standard deviation (minimum; maximum). Categorical variables are reported as frequency. Age and education were compared between groups using analysis of variance (ANOVA) models (*p* < 0.05) on rank-transformed data. Education-adjusted ANCOVA models (*p* < 0.05) on rank transformed data or χ2 test for categorical variables (*p* < 0.05) were used to compare groups. Bold font indicates significant results.

*HC* healthy control, *H&Y* Hoehn and Yahr score, *LEDD* levodopa equivalent daily dose, *mg* milligrams, *MAM* Manual Ability Measure questionnaire, *MDS-UPDRS-II or III* Movement Disorder Society Unified Parkinson’s Disease Rating scale part II or part III, *M/F* male/female, *MMSE* Mini-Mental State Examination, *N* Number, *PDQ-39* Parkinson’s Disease Questionnaire-39, *PPT* Purdue Pegboard Test, *pwPD* people with Parkinson’s Disease.

### Kinematic results (optical fiber glove)

During NOCue condition, pwPD showed a greater tendency to reduce movement amplitude with repetition (sequence effect) relative to healthy controls; while they showed slower movement during Cue condition.

In addition, uncorrected exploratory results showed that during NOCue condition, pwPD showed a smaller average movement amplitude relative to healthy controls.

**Fig. 1:**
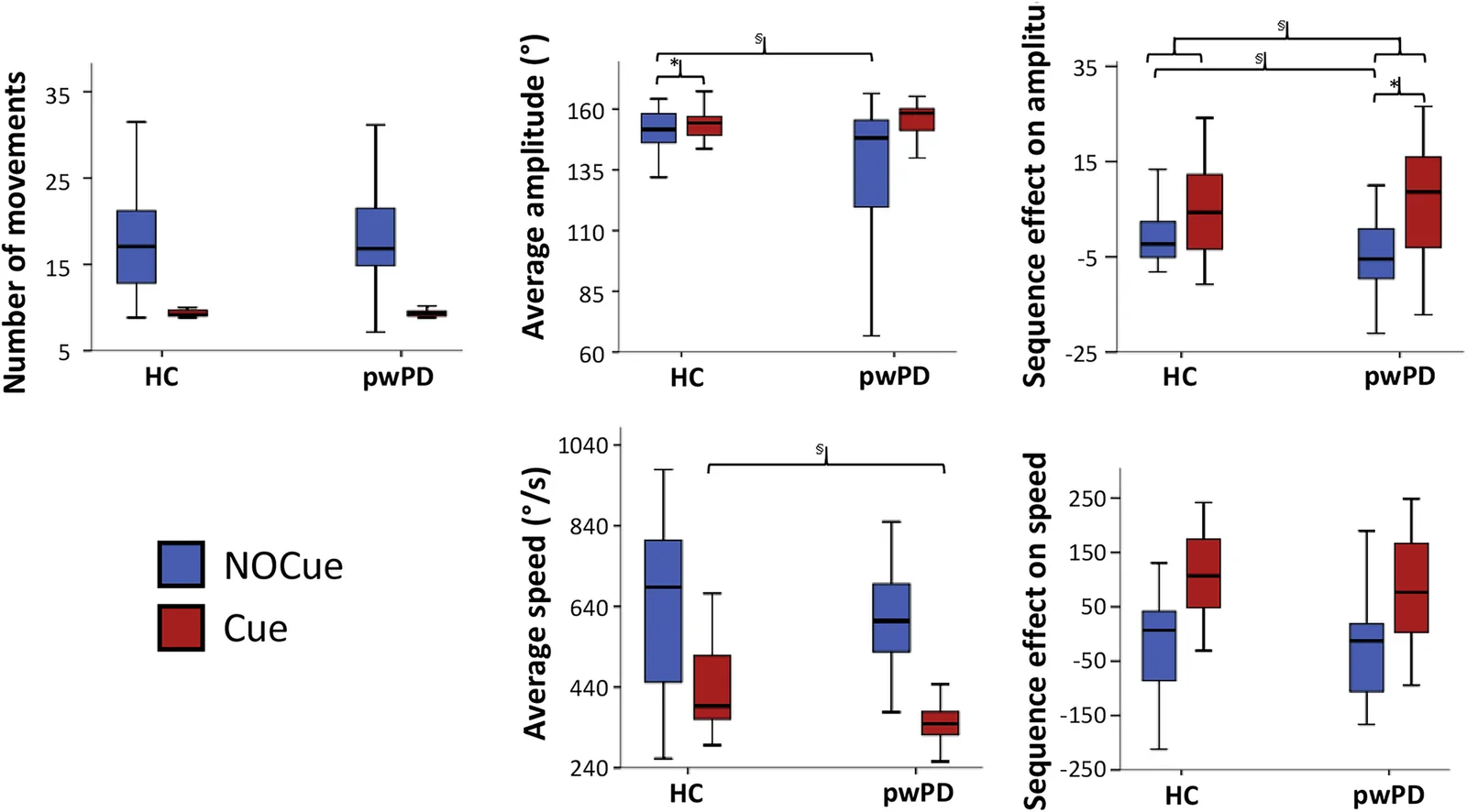
Kinematic parameters assessed with 5DT Data Glove during the fMRI hand-tapping task with the right hand with and without auditory cues (Cue/ NOCue) in pwPD and healthy controls. Box plot central line refers to median; box limits refer to first and third quartiles; whiskers refer to minimum and maximum values. * indicates significant within-group differences (Cue vs NOCue); ^§^ indicates significant between-group differences (pwPD vs healthy controls in the single conditions—Cue/NOCue or interaction group*condition). Statistical significance (uncorrected): *p* < 0.05. Higher (positive) sequence effect value indicates a tendency to increase amplitude/speed with movement repetition; lower (negative) sequence effect value indicates a tendency to reduce amplitude/speed with movement repetitions. HC healthy controls, pwPD people with Parkinson’s Disease, s seconds.

Healthy controls exhibited increased average hand-tapping amplitude during the Cue task relative to the NOCue task, while PwPD showed a lower tendency to reduce the movement amplitude with task repetition during Cue condition compared to the NOCue one. PwPD also displayed a larger improvement in sequence effect with cues than healthy controls (Fig. 1 and Supplementary Table 1).

### Functional MRI results

**Fig. 2:**
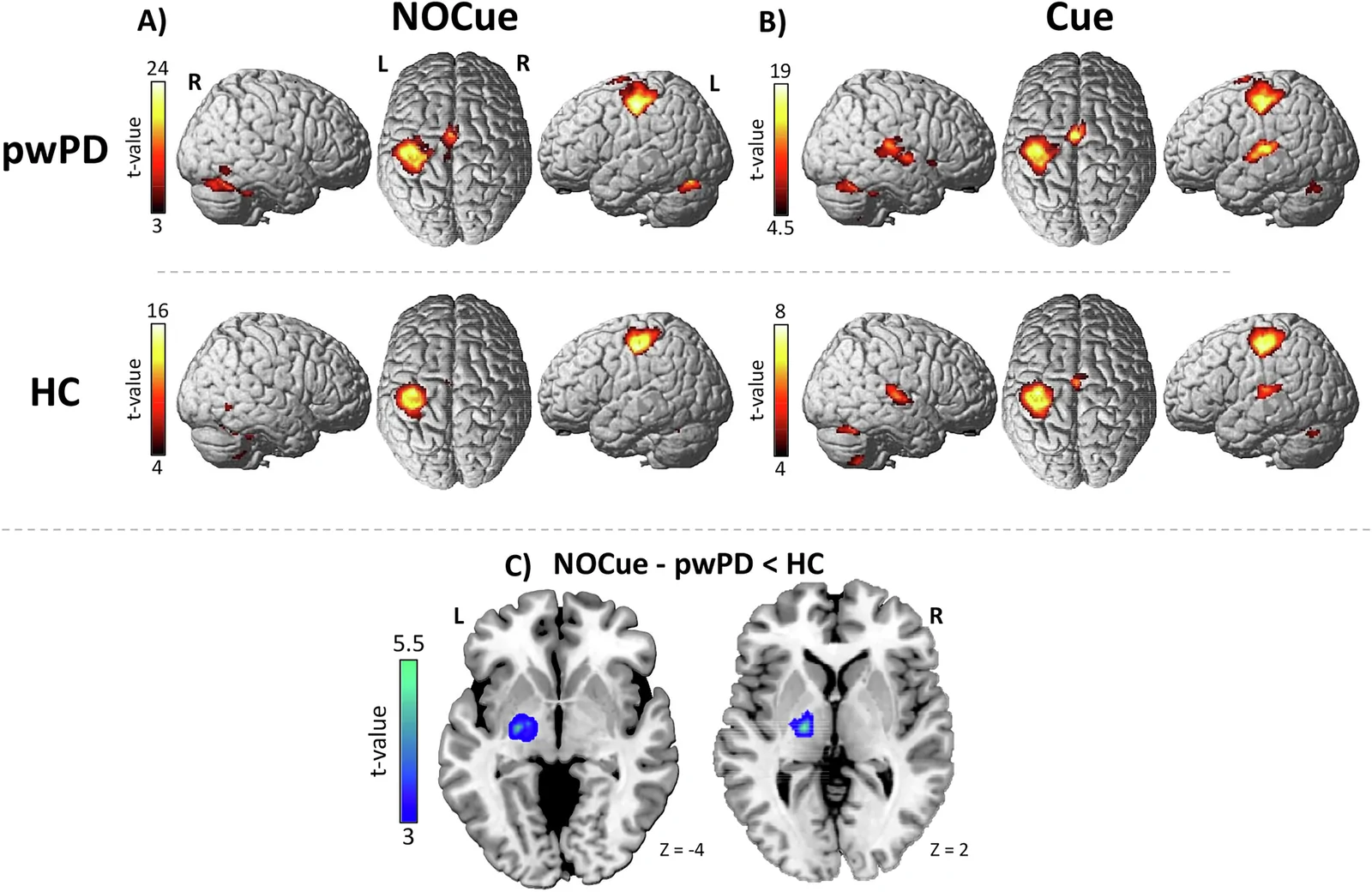
fMRI brain activity during the right hand tapping with (Cue) and without (NOCue) cueing tasks in pwPD and healthy controls. **A** Brain activity pattern during NOCue condition in pwPD and healthy controls. Results are shown on a render of the Montreal Neurological Institute (MNI) standard brain. Color bars denote t-values. **B** Brain activity pattern during Cue condition in pwPD and healthy controls. Results are shown on a render of the Montreal Neurological Institute (MNI) standard brain. Color bars denote t-values. **C** Comparison of brain activity in NOCue condition between pwPD and healthy controls. Results are shown on an axial section of the Montreal Neurological Institute (MNI) standard brain. Cold colors indicate decreased brain activity in pwPD relative to healthy controls.Findings shown are FWE corrected at voxel level (*p* < 0.05). Cue hand tapping with auditory cue, HC healthy controls, L left, NOCue hand tapping without auditory cue, pwPD people with Parkinson’s Disease. R right.

During the NOCue condition, both groups showed a similar pattern of activation including left primary motor cortex, basal ganglia and right cerebellum (Fig. 2A).

During the Cue condition, both healthy controls and pwPD demonstrated activation in temporal regions related to hearing in addition to movement-related areas (Fig. 2B).

When comparing the two groups, pwPD exhibited a reduced activation in the left pallidum (both internal and external portion) and thalamus compared to healthy controls during the NOCue task (Fig. 2C, Supplementary Table 2, and Supplementary Fig. 2).

We found no significant differences between pwPD and healthy controls during the Cue task.

**Fig. 3:**
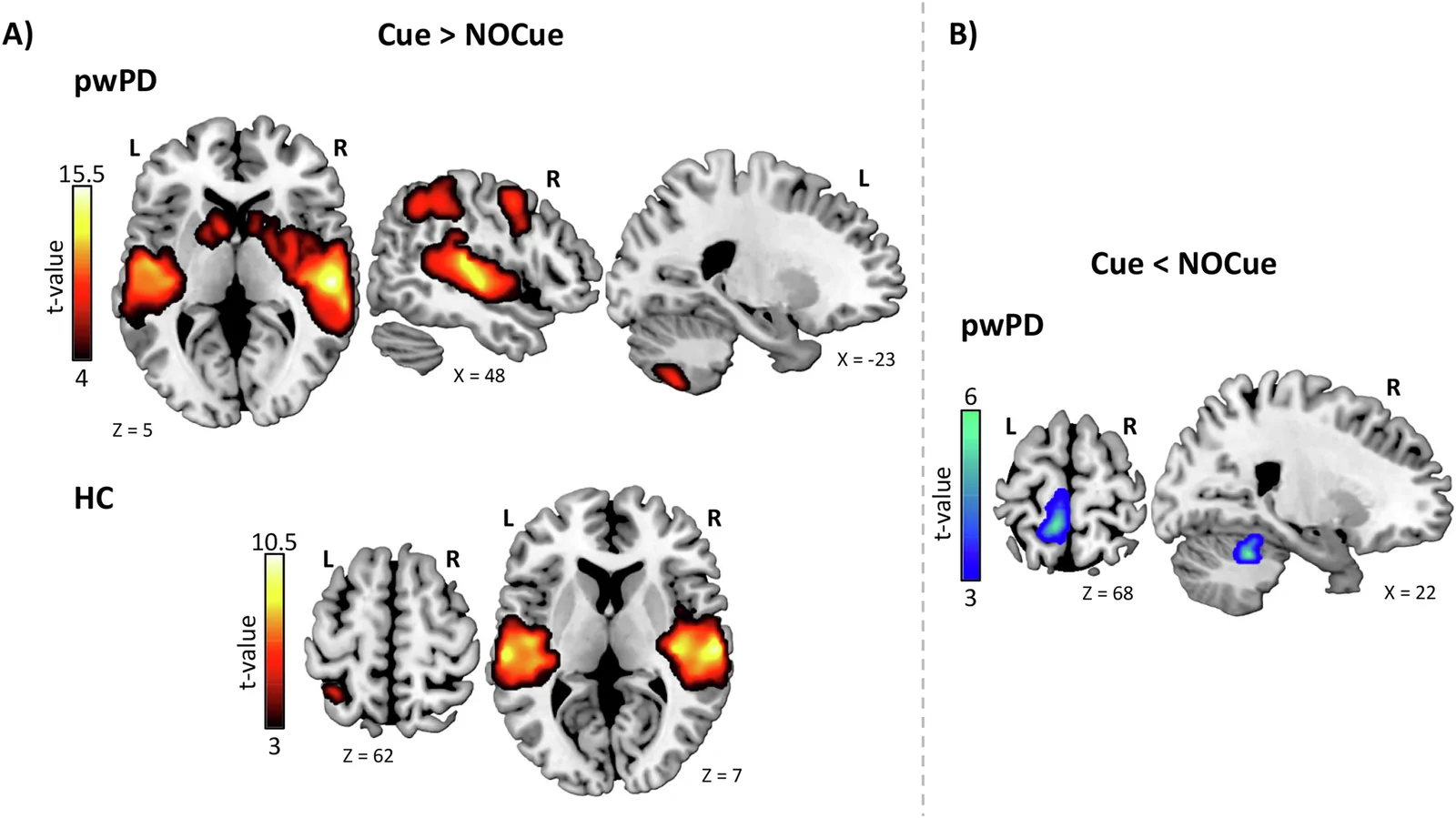
fMRI activation pattern during hand tapping task with auditory cue (Cue) relative to hand tapping without auditory cue (NOCue) in pwPD and healthy controls. **A** Areas of increased activity in Cue relative to NOCue condition in pwPD and healthy controls. **B** Areas of decreased activity in Cue relative to NOCue condition in pwPD.Results are shown on axial or sagittal section of the Montreal Neurological Institute (MNI) standard brain. Color bars denote t-values. Cluster colors indicate increased (warm colors) or decreased (cold colors) activity during Cue condition compared with NOCue condition. Findings shown are FWE corrected at voxel level (*p* < 0.05). Cue hand tapping with auditory cue, HC healthy controls, L left, NOCue hand tapping without auditory cue, pwPD people with Parkinson’s Disease, R right.

When comparing the two conditions (Cue vs NOCue), both groups demonstrated increased activation of the bilateral superior temporal gyrus, Heschl’s gyrus, and right insula during the Cue condition relative to the NOCue condition (Fig. 3A and Supplementary Table 3). Moreover, healthy controls showed increased activation of the left middle temporal and left superior parietal gyri in the Cue condition compared to the NOCue condition; whereas pwPD showed greater activation of the bilateral putamen, (external) pallidum, and caudate, as well as the right middle temporal, supramarginal, precentral and postcentral, and inferior parietal gyri, left insula and cerebellar lobule VIII (Fig. 3A, Supplementary Table 3, and Supplementary Fig. 2). PwPD also demonstrated a reduced activation of the left paracentral gyrus/supplementary motor area (SMA) and right cerebellar lobules IV-V-VI during Cue relative to NOCue (Fig. 3B and Supplementary Table 3).

### Correlations between kinematic and functional MRI data

Supplementary Table 4 reports areas of the Automated Anatomical Labeling (AAL) atlas overlapping with clusters of fMRI results. For correlation analyses, we extracted the mean t value from the following significant motor areas: thalamus, pallidum, precentral gyrus, and cerebellum IV-VI.

**Fig. 4:**
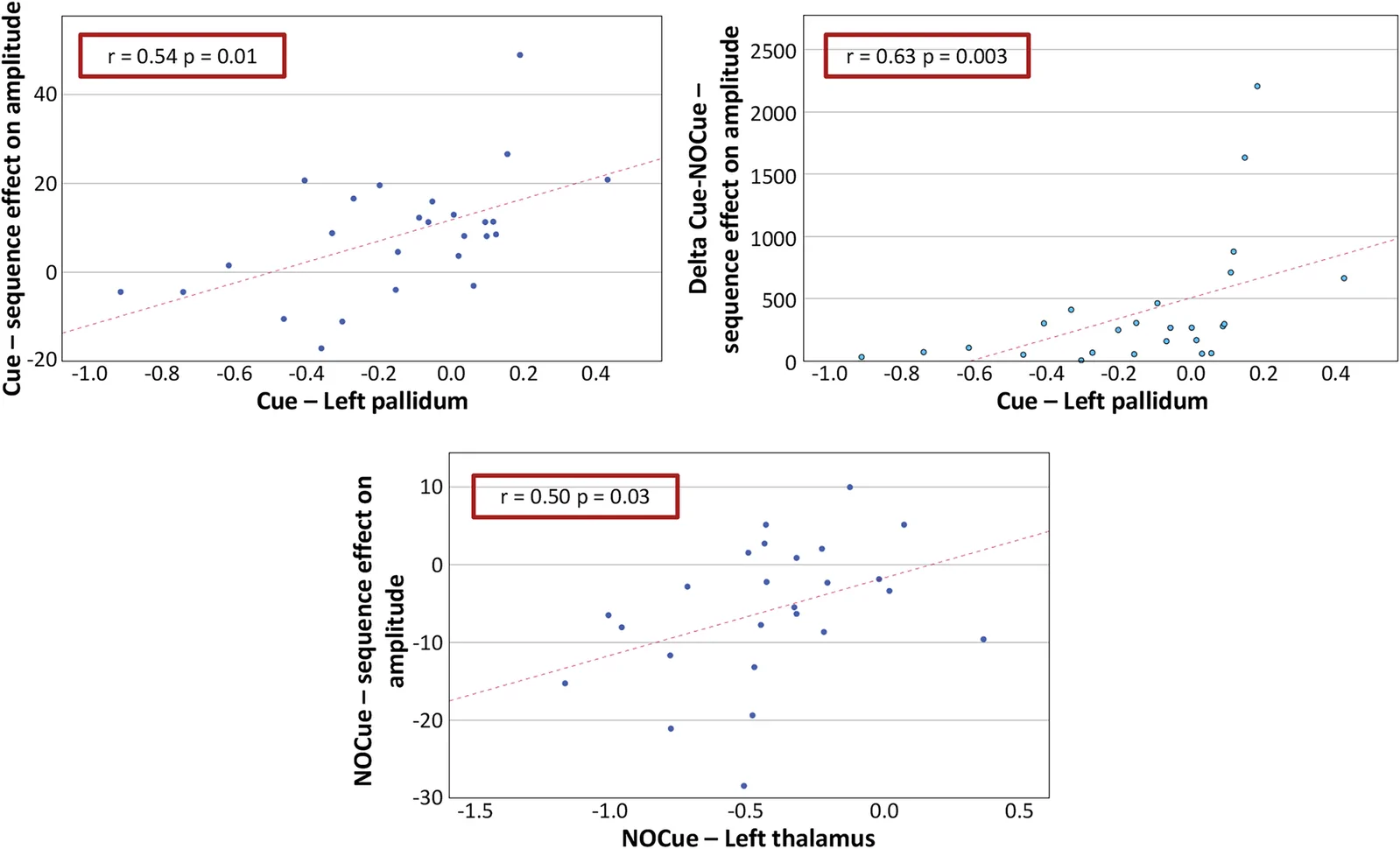
Correlations between fMRI activation and kinematic data during Cue and NOCue conditions in people with Parkinson’s disease. *p* and *r* values refer to Spearman correlations (*p* < 0.05) Bonferroni corrected for number of conditions (Cue, NOCue, Delta Cue-NOCue). The X-axes represent the task condition during the fMRI task and the associated activation cluster. The Y-axis represents the task condition and the kinematic parameter. Higher (positive) sequence effect value indicates a tendency to increase amplitude/speed with movement repetition; lower (negative) sequence effect value indicates a tendency to reduce amplitude/speed with movement repetitions. Cue hand tapping with auditory cue, NOCue hand tapping without auditory cue.

In pwPD, lower activation of the left thalamus during the NOCue task was associated with a higher tendency to reduce hand tapping amplitude during NOCue condition. During the Cue task, increased left pallidal activation correlated with a reduced tendency to diminish movement amplitude with task repetition (better sequence effect). A higher left pallidum activation in the Cue condition was also associated with a greater improvement of the sequence effect on amplitude in the Cue condition compared to the NOCue condition (Fig. 4 and Supplementary Table 4). No correlations were found between levodopa equivalent daily dose (LEDD) and fMRI or kinematic data.

## Discussion

In this study, we investigated the neural and kinematic correlates of repetitive hand tapping with and without auditory cues in pwPD using an MRI-compatible optic fiber glove that was previously used and validated^9,22,23^. The study included patients with early PD and with right upper limb motor involvement. We found that external auditory cues could modulate both movement performance and underlying brain activity in pwPD.

Consistent to what we previously showed, during self-paced tapping, pwPD patients exhibited movement alterations typical of the bradykinetic complex and particularly hypokinesia and sequence effect manifested as reduced movement amplitude and a greater tendency to progressively decrease amplitude with repetitions^2^.

At the neural level, pwPD and healthy controls showed a pattern of activation consistent with previous extensive evidence on brain regions associated with motor task performance, such as the primary sensorimotor cortex, SMA, basal ganglia, and cerebellum^14,24,25^.

However, during self-paced tapping, pwPD showed reduced activation of pallidum/thalamus relative to healthy controls, which was correlated with the reduced movement amplitude measured with the optical glove. Sequential movements highly depend on basal ganglia-cortical interaction as they require the correct implementation and control of movement amplitude and frequency^15^.

Specifically, pallidum is a key structure that contributes to the regulation of movement amplitude by modulating the inhibitory output of the basal ganglia toward thalamo-cortical motor circuits, thereby scaling motor commands and ensuring stable execution of sequential movements over time^26^. Under physiological conditions, the SMA and the basal ganglia form a functional loop that acts as an internal cueing system, in which continuous pacing-related adjustments are crucial for sustaining ongoing movements. With practice, this process becomes progressively automatized; however, it deteriorates in PD as a consequence of basal ganglia degeneration^16,27^.

Moving to the primary aim of this study, during hand tapping with auditory cueing, subjects revealed changes in both movement execution and sensorimotor neural processing. Indeed, both between- and within-group analyses revealed a significant reduction of the sequence effect in pwPD. Specifically, while during self-paced hand tapping they showed a progressive reduction of movement amplitude with repetition, during the cued task they tended to increase amplitude, similarly to healthy controls. This occurred despite a slight reduction in movement speed associated with following the imposed rhythm, which likely reflects a functional trade-off to maintain amplitude rather than a deficit in performance. This result aligns with the “stabilizing role” of cues proposed by Ginis and colleagues, suggesting that auditory cueing supports more appropriate timing and amplitude, stabilizing spatiotemporal parameters of movement^10,28^.

Moreover, during the Cue task, pwPD did not differ from healthy controls in either average movement amplitude or neural activity. We can speculate that external auditory pacing may compensate for impaired internal motor regulation by facilitating basal ganglia–sensorimotor processing and, consequently, motor output in pwPD^29–32^. However, this interpretation should be considered cautiously, as it is solely based on the absence of significant between-group differences (that does not imply equivalence).

As expected during a task involving hearing, similar to healthy subjects, pwPD exhibited increased activation in temporo-parietal regions involved in auditory processing. Previous studies have suggested that rhythmic auditory cues may exert network-level effects in pwPD, enhancing interactions between auditory and executive networks that, in turn, modulate cortico-cerebellar circuits^33^. Consistent with entrainment models, such auditory stimulation can synchronize and stabilize motor responses in pwPD^34^, possibly reducing another relevant aspect of the bradykinesia in pwPD that is dysrhythmia^35,36^. In support of this previous evidence, we found that during Cue condition relative to NOCue task, pwPD showed increased activity of basal ganglia, precentral gyrus, and motor part of cerebellum (lobule) VIII, which are key structures for sensorimotor integration, timing, and movement execution^28,37,38^. Interestingly, the increased activity of the pallidum was correlated with the reduced sequence effect on amplitude that we observed with cueing in pwPD supporting its stabilizing effect on movement. Increased activity of pallidum was mainly located in the external portion. The involvement of external globus pallidus is consistent with previous evidence showing that its activity is related to movement speed and amplitude, particularly during cued movements^39^.

Moreover, during the cued condition compared to self-paced tapping, pwPD showed decreased activity in the paracentral gyrus, SMA, and cerebellar lobules IV–VI, which may reflect a reduced demand for movement planning and a shift of motor control toward more efficient sensorimotor integration networks. This observation aligns with previous evidence: a study on the neural correlates of the sequence effect during handwriting in pwPD demonstrated a link between micrographia and dysfunction of basal ganglia motor circuits, coupled with reduced connectivity with the pre-SMA and cerebellum^40^. Similarly, a recent meta-analysis reported decreased SMA and cerebellar activity during finger tapping with auditory cues relative to self-paced tasks, supporting the idea that cueing may streamline motor execution by optimizing the recruitment of sensorimotor networks^14^.

A major strength of the study lies in the concurrent acquisition of fMRI data and detailed kinematic recordings of hand movements. This multimodal approach addresses a clinical limitation of most fMRI investigations in pwPD, which typically lack simultaneous high-resolution behavioral data^41^. To further study PD-associated brain pathophysiology, other MRI approaches like DTI or volumetry may be added in future studies to further correlate the activation patterns to microstructural/structural damage associated with the disease. The study also has some limitations. The sample size was relatively small, reflecting the inherent challenges of recruiting pwPD who can tolerate and successfully complete fMRI trials. Moreover, the inclusion criteria defined a specific sample of pwPD with right upper limb motor involvement, reducing generalizability of results. To maximize the robustness of our findings, fMRI results were reported using FWE correction. The enrolled participants were characterized by a relatively short disease duration (mean 4 years), which may have facilitated the detection of early features of the bradykinesia complex, such as the sequence effect^42^. At the same time, the ability to capture these subtle kinematic alterations in an early-stage cohort represents a major strength of the study. Future study could assess a pwPD with different characteristics and ideally also follow patients longitudinally to track how bradykinesia and its response to cues evolve with disease progression. To minimize medication effects on brain activity, patients were tested in the OFF state, nevertheless, given the ample range of LEDD in our sample and the influence that it could have on motor performance, we assessed the correlation between LEDD and kinematic and fMRI data and found no significant result. Future investigations should extend this paradigm to patients in the ON state and compare both conditions. Furthermore, the study focused exclusively on auditory cueing; investigating the effects of different types of cues (e.g., visual) or varying cue parameters (e.g., frequency) would offer a more comprehensive understanding of cueing effects. Finally, we only assessed the immediate effects of auditory cueing, so conclusions regarding long-term retention, generalization to complex tasks, or durability of neural changes cannot be drawn. Another limitation of the study is the fixed order of the tasks: the improvements could partly reflect repetition of the task rather than effects of cueing. However, the simplicity of the task and the familiarization before the acquisition limited possible learning effects. Moreover, the effects of fatigue were mitigated by the acquisition of other MRI sequences between the two tasks to provide some minutes of rest. A further limitation concerns the potential circularity in the brain-behavior correlation analyses, as the regions of interest and the kinematic variables derived from the task are not fully independent. We chose to use a hypothesis-driven approach and limit the correlations to data significantly different within/between-group to reduce the number of correlations and consequently the risk of false positives or confounding results.

In conclusion, these findings suggest that external auditory cues may modulate both motor performance and brain activity in pwPD. Specifically, during their application, cues might stabilize motor performance and counteract bradykinesia by maintaining movement amplitude. Possible mechanisms underlying these movement improvements may be a re-engagement of the pallidum/thalamus, reduced demand for movement planning, and more efficient recruitment of motor resources. This study provides additional insight into the mechanisms underlying cueing, reinforcing the idea that rhythmic auditory stimulation could be considered as tool to support motor control in rehabilitation settings.

## Methods

### Subjects and study design

This study is part of a randomized controlled trial that investigated the effects of physiotherapy on upper limb function in pwPD (ClinicalTrials.gov ID: NCT04876352). Right-handed outpatients with idiopathic PD^43^ were recruited at the Movement Disorder Unit, Neurology Department, IRCCS Ospedale San Raffaele, Milan, Italy, according to the following inclusion criteria: Hoehn & Yahr (H&Y) stage ≤3 while on medication; age ≤85 years; right-side motor involvement based on H&Y and MDS-UPDRS-III; and handwriting difficulties (MDS-UPDRS II.7 ≥ 1). Age- and sex-matched right-handed healthy controls were recruited through word of mouth among non-consanguineous relatives and institute staff.

Exclusion criteria for both pwPD and healthy controls included: Mini-Mental State Examination (MMSE) < 24 in pwPD or <28 in healthy controls; cerebrovascular disorders; a history of alcohol and/or psychotropic substance abuse; and failure to provide oral and written informed consent. All participants (pwPD and healthy controls) underwent dexterity and upper limb motor functional assessments, respectively with Purdue Pegboard Test (PPT) and Manual Ability Measure questionnaire (MAM-36), neuropsychological screening with the MMSE and brain MRI scans. PwPD also received a neurological assessment: an experienced neurologist administered H&Y staging and MDS-UPDRS parts II and III in both the ON and OFF medication states (the latter conducted at least 12 h after the regular evening dose of dopaminergic medication, or 24 h for dopamine agonists).

The study protocol was approved by the local ethics committee for human research (Ethical Committee IRCCS San Raffaele Scientific Institute, Milan, Italy; No. 68/int/2019), and written informed consent was obtained from all participants prior to inclusion in the study. All methods were performed in accordance with relevant guidelines and regulations, including the Declaration of Helsinki.

### MRI protocol

Using a 3.0 Tesla Philips Intera scanner (Ingenia CX, Philips Medical System, Best, The Netherlands), brain MRI scans were obtained during OFF time (at least 12 h after the regular evening dopaminergic therapy administration, except for dopamine agonists that required 24 h discontinuation), to mitigate the pharmacological effects on neural activity. Both functional and structural MRI sequences were acquired. The following structural brain MRI sequences were acquired to exclude subjects with eventual structural brain alterations and/or excessive vascular lesions: (i) 3D T2-weighted: repetition time (TR) = 2500 ms, echo time (TE) = 330 ms, flip angle = 90°, 192 contiguous sagittal sections, thickness = 1 mm, field of view (FOV) = 256 mm × 256 mm, matrix = 256 × 258, voxel reconstruction = 0.9 mm × 0.9 mm × 1 mm; (ii) 3DT1-weighted: TR = 7.1 ms, TE = 3.2 ms, flip angle = 9°, 204 contiguous sagittal sections, thickness = 1 mm, FOV = 256 mm × 240 mm, matrix = 256 × 240, voxel reconstruction = 1 mm × 1 mm × 1 mm; (iii) 3D Flair: TR = 4800 ms; TE = 269 ms; flip angle = 40°; 192 contiguous sagittal sections; thickness = 1.5 mm; FOV = 256 × 256 mm; matrix = 256 × 256, voxel reconstruction 1 × 1 × 1.

### Functional MRI study

FMRI was obtained using a T2* weighted echo planar imaging sequence with the following parameters: TE = 35 ms, TR = 1572 ms, flip angle = 70°, FOV = 240 × 240 mm, matrix = 96 × 94, 48 contiguous axial sections, thickness = 3 mm, voxel reconstruction 2.5 × 2.5 × 3 mm. During the fMRI hand tapping task, participants were instructed to open and close their right hand as ample and fast as possible for about 10 s (following the performance guidelines of MDS-UPDRS item 3.5) while wearing an MRI-compatible optical fiber glove (NOcue condition). Participants then performed the same maximal-amplitude hand tapping movements but following an external auditory cue (metronome with electronic beep) presented at 2 Hz (Cue condition). Patients were asked to open or close their right hand every time they heard a sound in the headphones. The 2 Hz represents an ideal frequency that allows pwPD to correctly react to each single cue^44^. A block design (ABAB) was used during the tasks: activation periods (A) corresponded to the execution of the hand tapping (six hand tapping periods lasting about 10 s each), while rest periods (B) represented resting periods without movements (six resting periods lasting about 10 s each). Subjects wore also an MRI-compatible headset which projected the commands to start and stop the task and a fixed cross during both the hand tapping movements and the resting period. Sandbags were used to position participants’ hands and prevent unwanted movements. Before entering the scanner, patients familiarized themselves with the hand movement required during the task both with and without cues to make sure they were able to correctly perform the requested movement in each condition.

### Optical fiber glove

As previously reported^9^, we employed the optical fiber 5DT Data Glove 14 Ultra (Fifth Dimension Technologies Inc., Orlando, USA), a one-size hand motion-capture device designed to measure finger flexion and abduction. Flexion of the fingers was detected at the metacarpal and proximal interphalangeal joints through 14 optical fiber sensors with a 12-bit A/D resolution (typically 10-bit range), integrated into a stretchable Lycra glove that ensures proper fitting. Fiber loops were connected to an LED, and the glove estimated finger flexion indirectly based on the amount of light returned to a phototransistor. The system was connected to a computer through a full-speed USB cable. A custom software application was developed specifically to record the glove’s signals.

### Hand tapping kinematic parameter analysis

Hand tapping kinematic parameters recorded with the optical fiber glove were processed using custom-made software. Each subject completed six hand-tapping periods for each condition. The software automatically identified amplitude peaks and counted the number of gestures based on the alternation between high peaks (open hand) and low peaks (closed hand), considering only movements with >20° excursion at the metacarpophalangeal joints. Peak detection was performed using a threshold-/crossing-based approach rather than derivative-based or template-matching methods. First, the raw signal was smoothed online by a moving average. Candidate movement segments were then identified from the intersections between the raw signal and its moving average, while enforcing a minimum temporal distance between consecutive crossings based on the estimated main frequency. Within each segment, local minima and maxima were extracted from the raw signal, and only segments showing sufficient excursion relative to the moving average were retained. Gestures were finally defined by pairing each peak with the preceding trough; very small movements were excluded using a minimum-amplitude threshold, and abnormally large events were removed as amplitude outliers. An operator visually inspected the amplitude and speed profiles for every hand tapping period on a computer screen to confirm that the automated detection procedure functioned properly. For each hand tapping period, the software extracted the number of hand tapping movements, the average amplitude and speed across all fingers, and the tendency to change movement speed and/or amplitude with repeated execution (sequence effect). Sequence effect was calculated as the slope of a linear regression fitted across successive movements, with movement time as the independent variable and amplitude (or speed) as the dependent variable. Parameters from the hand tapping periods in each condition were then averaged for the analyses.

### Functional MRI analysis

FMRI data were analyzed using SPM12 software (http://www.fil.ion.ucl.ac.uk/spm; Wellcome Trust Center for Neuroimaging, London, UK). Prior to statistical analysis, all images were realigned to the first one to correct for subject head motion (all study participants showed maximal head movements lower than 3 mm in each direction), slice-timing corrected, coregistered to subject’s 3DT1, spatially normalized into the standard MNI (Montreal Neurological Institute) space and smoothed applying a 8-mm 3-D Gaussian filter to improve signal-to-noise ratio and accommodate residual inter-subject anatomical variability following spatial normalization^45^. The signal variations of the BOLD effect associated with the execution of each task (considering head movement parameters as confounds) were evaluated voxel by voxel using the general linear model (GLM) and Gaussian field theory. Specific effects were tested applying appropriate linear contrasts. Significant hemodynamic changes for each contrast were evaluated using a nonparametric permutation-based approach, that is, Statistical non-Parametric Mapping (SnPM), a toolbox for SPM^46^.

One-sample *t* tests were used to assess the average fMRI activity during each task, while the paired t test was used to evaluate within group contrasts between Cue and NOCue conditions; differences between groups (healthy controls vs pwPD) were assessed using a two-sample *t* tests including education as covariate. Differences between the pwPD and healthy controls in Cue vs NOCue conditions were evaluated using a GLM model, in which group and condition were included as distinct factors (2 × 2 factorial design) with education as covariate.

To restrict the search space and reduce multiple comparisons, a mask was generated using the AAL atlas and implemented in SnPM via the WFU PickAtlas. The mask included the bilateral precentral and postcentral gyri, SMA, paracentral lobule, superior and inferior parietal cortices, caudate, putamen, pallidum, thalamus, insula, Heschl’s gyrus, superior/middle/inferior temporal cortices, and the cerebellum; these areas were selected as part of the sensorimotor network and involved in the processing of auditory information^33,47^. Supplementary Figure 1 reports the fMRI analyses results with the applied mask. For all permutation-based contrasts, nonparametric testing was performed with 5000 random permutations. Findings are shown FWE corrected at voxel level (*p* < 0.05). Only clusters exceeding 10 voxels were retained for interpretation.

We overlapped clusters of fMRI results to the AAL atlas to identify the motor areas showing significant differences in between-group (pwPD vs healthy controls) or within-pwPD (Cue vs NOCue). Mean t values were extracted from those motor regions to perform correlation analyses with kinematic data.

### Statistical analysis

Age and education were compared between groups using analysis of variance (ANOVA) models on rank-transformed data (*p* < 0.05). Given the different education between pwPD and healthy controls, education-adjusted ANCOVA models (*p* < 0.05) on rank-transformed data or χ2 test for categorical variables (*p* < 0.05) were used to compare clinical and kinematic data between groups. We reported both results surviving Bonferroni correction for number of groups, and uncorrected results for exploratory interpretations.

General linear models for repeated measures on rank-transformed values were used to compare the effect of auditory cues within group; the same models, with education as covariate, were used to compare the effects of auditory cues between groups (interaction group*condition).

Spearman’s rank correlation coefficient was used for correlation analyses between significant fMRI and kinematic results, correlation analyses were Bonferroni corrected for number of conditions (Cue, NOCue, Delta Cue-NOCue). All statistical analysis were performed using IBM SPSS Statistics 30.0 software.

## Supplementary information

Supplementary materials_R1_Clean.

## Data Availability

The dataset used and analyzed in this study is deposited in the San Raffaele Open Research Data Repository (ORDR), an institutional platform powered by Digital Commons Data that enables the storage, preservation, and sharing of research data. The repository is accessible through the following https://doi.org/10.17632/6nygwkbrjf.1 The scripts for kinematic data extraction and fMRI analyses are available at https://github.com/SilviaBasaia/Auditory-cueing-modulates-motor-performance-and-brain-activity-in-early-Parkinson-s-disease-.
