# Supplementary material for: Auditory cueing modulates motor performance and brain activity in early Parkinson’s disease—a kinematic and fMRI study
Source: NPJ Parkinsons Dis. 2026 Jun 19;12:243. doi: 10.1038/s41531-026-01438-0 (PMC13639042; doi:10.1038/s41531-026-01438-0)
Supplement: Supplementary file 1 [file 41531_2026_1438_MOESM1_ESM.pdf]

Supplementary table 1 - Right hand tapping kinematic data with (Cue) and without (NOCue) auditory cues in people with Parkinson's disease and healthy controls.

	**pWD**	**HC**	**p pwPD vs HC**
**NOCue – Number of movements**	18.19 ± 6.56 (7.17; 37.83)	17.47 ± 6.21 (8.83; 31.50)	0.39
**Cue – Number of movements**	9.30 ± 0.54 (7.33; 10.17)	9.36 ± 0.39 (8.83; 10.00)	1.00
**p Cue vs NOCue**	0.54*	0.75*	0.75§
**NOCue – Average amplitude [°]**	136.45 ± 29.29 (57.78; 166.48)	149.62 ± 11.24 (119.02; 164.22)	**0.03**
**Cue – Average amplitude [°]**	154.19 ± 10.88 (118.13; 165.31)	153.63 ± 7.81 (132.77; 167.31)	0.97
**p Cue vs NOCue**	0.69*	**0.03***	0.09§
**NOCue – Sequence effect on amplitude**	-5.87 ± 8.94 (-28.46; 9.97)	-0.95 ± 5.33 (-8.16; 13.35)	**<u>0.01</u>**
**Cue – Sequence effect on amplitude**	8.33 ± 13.68 (-17.17; 48.91)	7.13 ± 14.21 (-10.78; 50.82)	0.68
**p Cue vs NOCue**	**0.04***	0.22*	**0.04**§
**NOCue – Average speed [°/s]**	602.79 ± 160.32 (229.27; 849.57)	657.10 ± 220.34 (262.78; 979.13)	0.59
**Cue – Average speed [°/s]**	359.02 ± 75.75 (192.99; 513.49)	427.70 ± 102.63 (296.07; 672.13)	**<u>0.02</u>**
**p Cue vs NOCue**	0.34*	0.30*	0.13§
**NOcue – Sequence effect on speed**	-34.52 ± 115.65 (-433.60; 189.66)	-17.93 ± 91.25 (-211.89; 130.48)	0.10
**Cue – Sequence effect on speed**	71.34 ± 94.50 (-94.25; 248.81)	102.06 ± 103.50 (-181.82; 241.99)	0.12
**p Cue vs NOCue**	0.69*	0.78*	0.98§

Values are mean ± standard deviation (minimum; maximum). Education-adjusted ANCOVA models ($p < 0.05$) on rank transformed data were used to compare pwPD and HC in the single conditions (Cue or NOCue). General linear models for repeated measures on rank transformed values were used to compare the effect of auditory cues within group (*); the same models, with education as covariate, were used to compare the effects of auditory cues between groups (interaction group*condition) (§). Bold font indicates significant results. Underlined font indicates results surviving Bonferroni correction for number of groups. Higher (positive) sequence effect value indicates a tendency to increase amplitude/speed with movement repetition; lower (negative) sequence effect value indicates a tendency to reduce amplitude/speed with movement repetitions. **Abbreviations**: HC = healthy control; pwPD = people with Parkinson's Disease.

Supplementary table 2 – fMRI pattern of activation in pwPD relative to healthy controls during hand tapping task with and without auditory cues performed with the right hand

**HAND TAPPING TASK (NOCue)**						
**pwPD < HC**						
**Ke**	**T**	**mni X**	**mni Y**	**mni Z**	**BA**	**Area**
15	5.37	-22	-10	-4	-	Left pallidum
		-18	-13	2	-	Left thalamus
**pwPD > HC**						
No significant clusters						
**HAND TAPPING TASK WITH AUDITORY CUE (Cue)**						
**pwPD < HC**						
No significant clusters						
**pwPD > HC**						
No significant clusters						

X, Y and Z coordinates refer to the Montreal Neurological Institute (MNI) space. Table reports results of the two-sample t-test performed in SnPM with 5000 random permutations ($p < 0.05$ FWE corrected at voxel level). Only clusters greater than 10 voxels are reported. **Abbreviations:** BA = Brodmann Area; HC = healthy controls; Ke = cluster extension; mni = Montreal Neurological Institute; pwPD = people with Parkinson's Disease.

Supplementary table 3 – fMRI pattern of activation during hand tapping task with auditory cue (Cue) relative to hand tapping without auditory cue (NOCue) in pwPD and healthy controls

**HC**						
**Cue > NOCue**						
**Ke**	**T**	**mni X**	**mni Y**	**mni Z**	**BA**	**Area**
1830	10.41	-44	-16	2	41	Left superior temporal
	8.91	-56	-22	0	21	Left middle temporal
	5.85	-48	-17	9	41	Left Heschl
2149	10.31	62	-20	10	41	Right superior temporal
	7.72	41	-20	6	41	Right Heschl
	4.65	48	2	0	13	Right insula
29	5.93	-34	-50	62	7	Left superior parietal
**Cue < NOCue**						
No significant clusters						
**pwPD**						
**Cue > NOCue**						
**Ke**	**T**	**mni X**	**mni Y**	**mni Z**	**BA**	**Area**
3226	15.49	58	-20	12	41	Right superior temporal
	8.75	59	-32	3	21	Right middle temporal
	8.39	41	-20	6	41	Right Heschl
	7.76	39	-18	6	13	Right insula
	4.57	29	-2	7	-	Right putamen
	4.04	21	-3	6	-	Right pallidum
	4.02	11	15	2	-	Right caudate
2267	13.99	-52	-20	14	41	Left superior temporal
	9.10	-38	-23	7	41	Left Heschl
	5.08	-40	-12	7	13	Left insula
369	10.14	48	4	44	6	Right precentral
144	7.31	-22	-62	-54	-	Left cerebellum VIII
755	6.31	50	-50	42	39	Right inferior parietal
	5.49	46	-40	44	40	Right supramarginal
	4.38	35	-30	47	1	Right postcentral
154	5.51	-9	10	4	-	Left caudate
	3.96	-16	5	4	-	Left pallidum
	3.93	-18	6	7	-	Left putamen
**Cue < NOCue**						
**Ke**	**T**	**mni X**	**mni Y**	**mni Z**	**BA**	**Area**
327	5.58	-8	-24	74	4	Left paracentral/SMA
58	5.21	20	-47	-26	-	Right cerebellum IV-V
	4.53	20	-51	-26	-	Right cerebellum VI

X, Y and Z coordinates refer to the Montreal Neurological Institute (MNI) space. Table reports results of the two-sample t-test performed in SnPM with 5000 random permutations ($p < 0.05$ FWE corrected at voxel level). Only clusters greater than 10 voxels are reported. **Abbreviations:** BA = Brodmann Area; HC = healthy controls; Ke = cluster extension; mni = Montreal Neurological Institute; pwPD = people with Parkinson's Disease, SMA = supplementary motor area.

Supplementary table 4 - Correlations between fMRI and kinematic data obtained during hand-tapping task with and without cue in pwPD.

p	r	+/-	Kinematic data - task	Mean fMRI activity - task
0.03	0.50	+	Average amplitude – NOCue (pwPD < HC)	Left thalamus – NOCue (pwPD < HC)
0.01	0.54	+	Sequence effect on amplitude – Cue (pwPD: Cue better than NOCue)	Left pallidum – Cue (pwPD: Cue > NOCue)
0.003	0.63	+	Sequence effect on amplitude – delta Cue-NOCue (pwPD: Cue better than NOCue)	Left pallidum – Cue (pwPD: Cue > NOCue)

p and r refer to Spearman's correlation Bonferroni corrected for number of conditions (NOcue, Cue, Delta Cue-NOCue). Positive correlation (+) means that both fMRI brain activity and kinematic value decrease or increase; negative correlation (-) means that and increase in fMRI brain activity is related to a decrease in the kinematic parameter (or vice-versa). "Better" sequence effect indicates a lower tendency to reduce amplitude with movement repetition. **Abbreviations:** +/- = positive/negative correlation; Cue = hand tapping with auditory cue; HC = healthy controls; NOCue = hand tapping without auditory cue; pwPD = people with Parkinson's Disease.

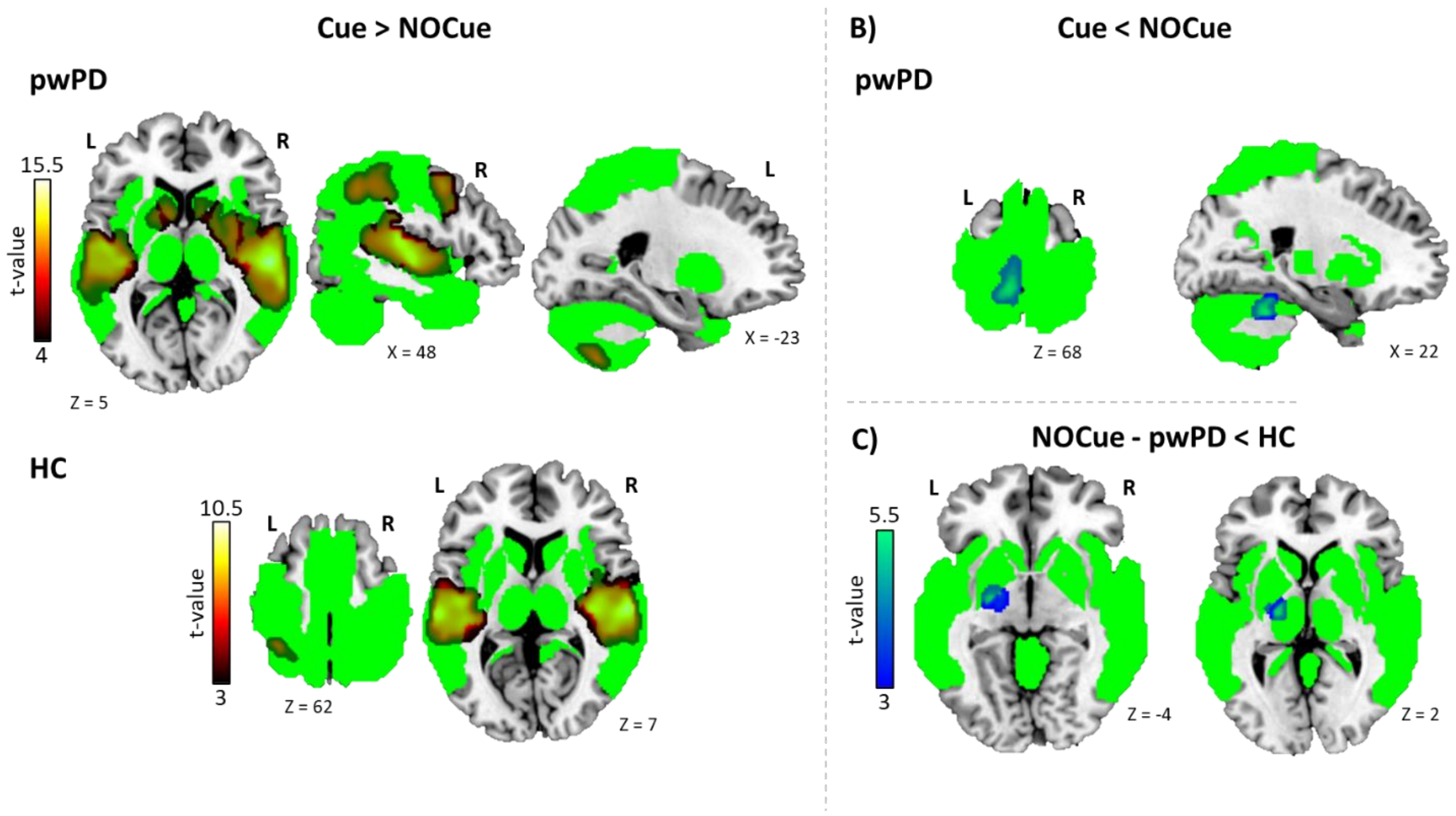

Supplementary figure 1 – Clusters of fMRI results (hot and cold colors) and brain mask used in the analyses (green). fMRI results are the same reported in Figures 2 and 3. The mask included the bilateral precentral and postcentral gyri, SMA, paracentral lobule, superior and inferior parietal cortices, caudate, putamen, pallidum, thalamus, insula, Heschl's gyrus, superior/middle/inferior temporal cortices, and the cerebellum; these areas were selected as part of the sensorimotor network and involved in the processing of auditory information.

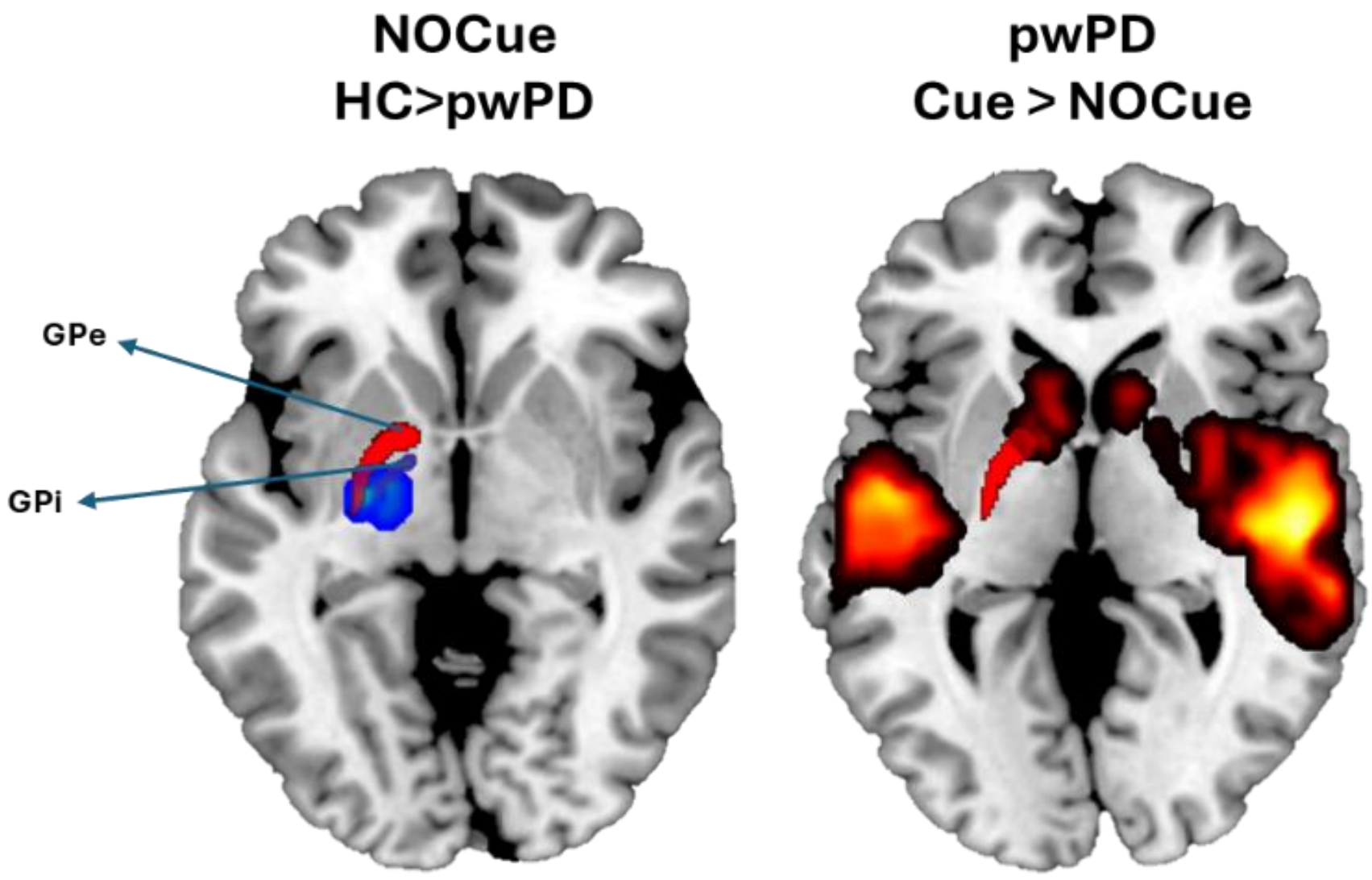

Supplementary figure 2 – Clusters of fMRI results (hot and cold colors) and mask of internal and external globus pallidus (GPi and GPe respectively). fMRI results are the one reported in Figures 2 and 3 overlapping with globus pallidus. **Abbreviations:** HC = healthy controls; pwPD= people with Parkinson's disease; GPi/e = internal/external globus pallidus.
